# On the use of antibiotics to control plant pathogenic bacteria: a genetic and genomic perspective

**DOI:** 10.3389/fmicb.2023.1221478

**Published:** 2023-06-27

**Authors:** Marie Verhaegen, Thomas Bergot, Ernesto Liebana, Giuseppe Stancanelli, Franz Streissl, Marie-Paule Mingeot-Leclercq, Jacques Mahillon, Claude Bragard

**Affiliations:** ^1^Laboratory of Food and Environmental Microbiology, Earth and Life Institute, Catholic University of Louvain (UCLouvain), Louvain-la-Neuve, Belgium; ^2^European Food Safety Authority (EFSA), Parma, Italy; ^3^Cellular and Molecular Pharmacology Unit, Louvain Drug Research Institute, UCLouvain, Woluwe-Saint-Lambert, Belgium; ^4^Plant Health Laboratory, Earth and Life Institute, UCLouvain, Louvain-la-Neuve, Belgium

**Keywords:** antibiotic resistance, *Erwinia amylovora*, horizontal gene transfer, one health, plant pathogenic bacteria, *strA-strB*, streptomycin, Tn*5393*

## Abstract

Despite growing attention, antibiotics (such as streptomycin, oxytetracycline or kasugamycin) are still used worldwide for the control of major bacterial plant diseases. This raises concerns on their potential, yet unknown impact on antibiotic and multidrug resistances and the spread of their genetic determinants among bacterial pathogens. Antibiotic resistance genes (ARGs) have been identified in plant pathogenic bacteria (PPB), with streptomycin resistance genes being the most commonly reported. Therefore, the contribution of mobile genetic elements (MGEs) to their spread among PPB, as well as their ability to transfer to other bacteria, need to be further explored. The only well-documented example of ARGs vector in PPB, Tn*5393* and its highly similar variants (carrying streptomycin resistance genes), is concerning because of its presence outside PPB, in *Salmonella enterica* and *Klebsiella pneumoniae*, two major human pathogens. Although its structure among PPB is still relatively simple, in human- and animal-associated bacteria, Tn*5393* has evolved into complex associations with other MGEs and ARGs. This review sheds light on ARGs and MGEs associated with PPB, but also investigates the potential role of antibiotic use in resistance selection in plant-associated bacteria.

## 1. Introduction

Plant pathogenic bacteria (PPB) cause devastating losses of crops worldwide, notably in vineyards, pear and apple orchards, estimated from one billion dollars every year (Mansfield et al., 2012; Kannan et al., 2015) up to five billion euros (Sánchez et al., 2019; Schneider et al., 2020). To cite a few, PPB such as *Erwinia amylovora* (affecting mainly pear orchards), *Xanthomonas oryzae* (damaging rice cultures), *Pseudomonas syringae* pv. *actinidiae* (harmful to kiwi plantations) or *Ralstonia solanacearum* (impacting tomato yields) represent major concerns. Emerging bacterial plant diseases such as *Candidatus* Liberibacter sp. (also known as Huanglongbing on citrus) or *Xylella fastidiosa*, which is the causal agent of Pierce’s disease of grapevine, citrus variegated chlorosis, olive quick decline syndrome and many other plant diseases, are growing threats. Therefore, on a worldwide scale, farmers often resort to antibiotics as a simple and effective tool for the control of bacterial diseases.

Although the amount of antibiotics used in plant protection is considered to be very low compared to human and veterinary medicine (McGhee and Sundin, 2011; McManus, 2014; FAO, OIE, and WHO 2018; Sundin and Wang, 2018), it has been suggested that their use may be more widespread than previously thought (McManus et al., 2002; McManus, 2014; O’Neill, 2015; Taylor and Reeder, 2020). The amounts effectively applied on crops are difficult to assess due to the lack of precise monitoring in different parts of the world. Currently, antibiotics authorized as plant protection products (PPPs) are commonly grouped with fungicides, e.g., in the Food and Agriculture Organization (FAO) statistics, because specific antibiotics can also be antifungal agents. Presently, different legislations, such as in Europe and West Africa, do not authorize antibiotics as PPPs, while their use is, to some extent, allowed in the American and Asian continents. Five antibiotics are most regularly reported to be used in plant agriculture: streptomycin (the most used antibiotic worldwide), oxytetracycline, kasugamycin, oxolinic acid (OA) and gentamicin (McManus, 2014; Sundin and Wang, 2018; Miller et al., 2022).

The application of antibiotics to plants exerts selective pressure on plant-associated bacteria, which can lead to the development of antibiotic resistance. Four main mechanisms result in bacterial antibiotic resistances: (i) inactivation of the antibiotic itself, (ii) reduction of its penetration (e.g., alteration of the cell membrane resulting in decreased permeability) or active elimination via efflux pumps, (iii) modification of the antibiotic targets, and (iv) use of alternative pathways [for reviews on antibiotic resistance mechanisms, refer to (Alekshun and Levy, 2007; Van Hoek et al., 2011; Blair et al., 2015; Munita and Arias, 2016)]. Bacteria have a high potential to acquire resistance to antibiotics, either through chromosomal mutations or *via* horizontal gene transfer (HGT) of antibiotic resistance genes (ARGs).

On the one hand, spontaneous gene mutations associated with the mechanism of action of the antibiotic can lead to cell survival. A resistant subpopulation can emerge and become prominent as the initial susceptible population will die (Munita and Arias, 2016). On the other hand, genetic material can be horizontally transferred through three main mechanisms (Dagan, 2011; Gyles and Boerlin, 2014): (i) transformation, which happens when a recipient cell is able to integrate free DNA found in the extracellular medium, (ii) transduction, where a bacteriophage (a bacterial virus) is involved in the gene transfer, and (iii) conjugation, that requires direct contact between two living cells for the exchange of genetic material. Mobile genetic elements (MGEs) can serve as vectors for the transfer of genetic material, either as intracellular, moving from one location to another within the same genome (e.g., insertion sequences or transposons), or intercellular elements, transferred from one cell to another (e.g., conjugative plasmids or integrative and conjugative elements, aka ICEs; Partridge et al., 2018).

The extent to which the use of antibiotics in plant agriculture could impact the global antibiotic resistance issue is unknown. The present review focuses on ARGs and MGEs associated with PPB. It highlights the potential correlation between the use of antibiotics against bacterial plant pathogens and the appearance of resistance. It also gathers critical information to be taken into account for risk assessments performed during the authorization process of an antibiotic for plant protection purposes.

## 2. Antibiotics used to control plant pathogenic bacteria

There are five major antibiotics most commonly used throughout the world against PPB (streptomycin, oxytetracycline, kasugamycin, oxolinic acid and gentamicin), although at least 15 have been identified as being used against plant diseases (Table 1; McManus, 2014; Sundin and Wang, 2018; Taylor and Reeder, 2020; Miller et al., 2022). They belong to eight different classes of antibiotics but the most prevalent are the aminoglycosides and β-lactams (Table 1). Among the top five, only kasugamycin is not used in human nor in veterinary medicine (Kumar et al., 2005; Aarestrup et al., 2008; McGhee and Sundin, 2011; McManus, 2014; World Health Organization, 2019). This is also the case of other antibiotics, such as ningnanmycin, validamycin or zhongshengmycin, used in China. Potentially, more antibiotics could be used in plant protection, but their use is not well monitored and/or their efficacy has not been demonstrated. In addition, in some parts of the world, antibiotics are applied to plants even though they are not authorized, due to poor control of antibiotic sales and/or lack of knowledge from producers (Chanvatik et al., 2019). Besides, there is a constant search for new antibiotics to fight plant diseases; for instance, penicillin shows great promises to combat citrus greening (Supplementary Table S1; Shin et al., 2016).

**Table 1:** Antibiotics used as PPPs, as of January 2023.

Antibiotics used as PPPs				
Class of antibiotic	Antibiotic	Countries or region	PPB	Reference(s)
Aminoglycosides	Gentamicin	Chile, Costa Rica, El Salvador, Honduras, Guatemala, Mexico	*Clavibacter michiganensis* ssp. *michiganensis, Erwinia amylovora, Pectobacterium carotovorum, Pectobacterium* spp., *Pseudomonas syringae* pv. *tomato, Pseudomonas* spp., *Ralstonia solanacearum, Ralstonia* spp., *Xanthomonas campestris* pv. *campestris*, *X. campestris* pv. *vesicatoria*, *Xanthomonas* spp.	Vidaver (2002), Rodríguez et al. (2006), Stockwell and Duffy (2012), Sundin and Wang (2018), Taylor and Reeder (2020), and Miller et al. (2022)
	Kasugamycin	Brazil, Canada, Japan, United States	*Acidovorax avenae, Burkholderia glumae, E. amylovora*, *P. syringae* pv. *garcae*, *Xanthomonas oryzae* pv*. oryzae*	Yashiro and McManus (2012), Barbosa et al. (2018), Sundin and Wang (2018), and Taylor and Reeder (2020)
	Streptomycin	Canada, Chile, China, Costa Rica, Hungary, Israel, Mexico, New Zealand, South Korea, Switzerland, United States	*Candidatus* Liberibacter sp., *C. michiganensis, C. michiganensis* ssp. *michiganensis, E. amylovora, P. syringae* pv. *actinidiae*	McManus et al. (2002), Németh (2004), Rodríguez et al. (2006), Stockwell and Duffy (2012), Cameron and Sarojini (2014), Walsh et al. (2014), Sundin and Wang (2018), Lyu et al. (2019), McVay et al. (2019), Valenzuela et al. (2019), Vincent et al. (2019), Hijaz et al. (2020, 2021), Killiny et al. (2020), Lee et al. (2020), Taylor and Reeder (2020), and Miller et al. (2022)
	Validamycin	China	*X. oryzae* pv. *oryzae*	Bian et al. (2020) and Taylor and Reeder (2020)
	Zhongshengmycin	China	*X. oryzae* pv. *oryzae*	Wang Q. et al. (2021)
Macrolides	Aureofungin	South East Asia	NA	Taylor and Reeder (2020)
Nucleosides	Ningnanmycin	Western Pacific	NA	Taylor and Reeder (2020)
Quinolones	Oxolinic acid	Israel, Western Pacific	*E. amylovora*	McManus et al. (2002), Stockwell and Duffy (2012), Sundin and Wang (2018), Taylor and Reeder (2020), Dafny-Yelin et al. (2021), and Miller et al. (2022)
Tetracyclines	Oxytetracycline	Brazil, Costa Rica, Mexico, United States	*Ca.* Liberibacter sp., *E. amylovora*, *Pectobacterium* spp., *Pseudomonas* spp., *X. campestris* pv. *viticola, Xanthomonas* spp.	McManus et al. (2002), Rodríguez et al. (2006), Stockwell and Duffy (2012), Naue et al. (2014), Sundin and Wang (2018), McVay et al. (2019), Vincent et al. (2019), Hijaz et al. (2020), Killiny et al. (2020), Taylor and Reeder (2020), Hijaz et al. (2021), and Miller et al. (2022)
	Tetracycline	India, Thailand	*Ca.* Liberibacter sp., *Candidatus* Phytoplasma sp.	McManus et al. (2002), Sundin and Wang (2018), Chanvatik et al. (2019), Taylor and Reeder (2020), and Rao (2021)
Thiadiazol	Bismerthiazol	China	*X. oryzae* pv. *oryzae*	Yang et al. (2021)
β-lactams	Amoxicillin	Thailand	*Ca.* Liberibacter sp.	Chanvatik et al. (2019) and Taylor and Reeder (2020)
	Ampicillin	Thailand	*Ca.* Liberibacter sp.	Chanvatik et al. (2019)
	Cefadroxil	America	NA	Taylor and Reeder (2020)
	Penicillins	Thailand	*Ca.* Liberibacter sp.	Chanvatik et al. (2019)

NA, information not available. A selection of antibiotics tested as PPPs can be found in Supplementary Table S1.

**Figure 1:**
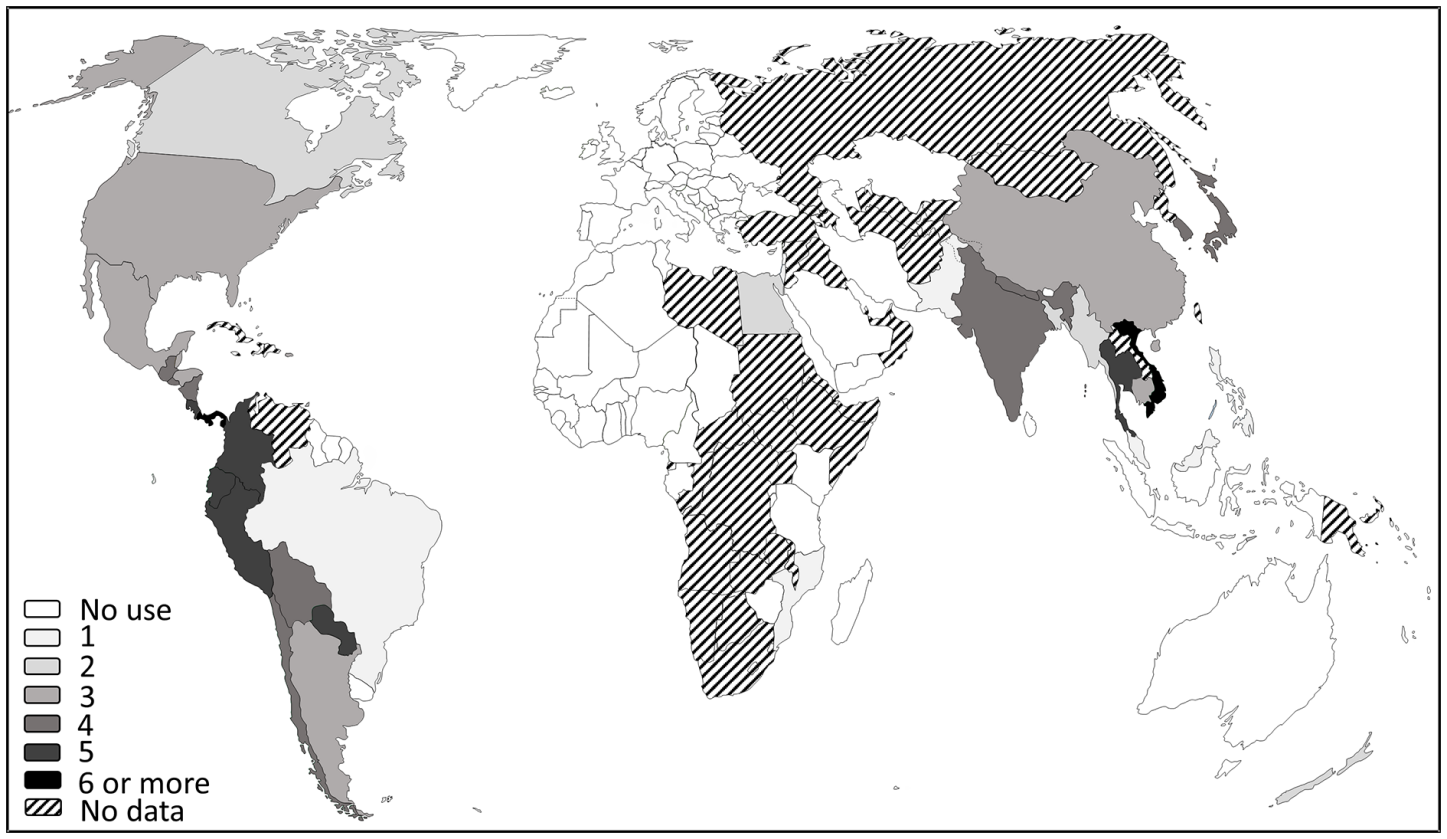
World map of countries where antibiotics are used as PPPs, compiled from scientific and grey literature searches screening the official list of authorized pesticides, when available, as of July 20, 2022 (list can be found in Supplementary Table S2). One hundred and ninety-five countries were considered, references could be found for more than 100 countries with around 70 with a clear indication that antibiotic use is not authorized in plants and around 30 countries with an indication of authorization. Numbers refer to the number of antibiotics used in each country. These data must be used with caution since legislations are subjected to rapid changes.

Globally, the use of antibiotics is permitted in the American and Asian continents but currently not approved in Europe (even if some limited derogations were accepted in the past) and West Africa (Figure 1; Supplementary Table S2). However, it is rather difficult to accurately list countries authorizing their use as PPPs, not only because available data are limited (Table 1), but also because the official lists of authorized pesticides are often available in the country’s official language only. Moreover, many countries suffer from a lack of monitoring of the use of antibiotics on plants. In fact, to the best of our knowledge, only three countries release information on the amounts used on crops: the United States, New Zealand and India.

## 3. Antibiotic resistance associated with plant pathogenic bacteria

Several ARGs have been identified in PPB, mainly for the five main antibiotics used in plant protection. Also, the best documented ARGs are the streptomycin resistance genes, while the mechanisms involved in the other resistances are less known (Table 2; McManus, 2014; Sundin and Wang, 2018; Miller et al., 2022).

**Table 2:** Antibiotic resistance mechanisms found in PPB, associated to the antibiotics used as PPPs.

Antibiotic	Class of antibiotic (general mode of action)	ARG(s) or gene modification leading to resistance	Type of resistance	Antibiotic resistance phenotype	Reference(s)
Gentamicin	Aminoglycosides (protein synthesis inhibitors)	*aacA3*	Gene acquisition (antibiotic inactivation)	*aacA3* encodes the aminoglycoside-3’-*N*-acetyltransferase, which inactivates gentamicin	Xu et al. (2013)
Kasugamycin	Aminoglycosides	*aac(2′)-IIa* (acyltransferase)	Gene acquisition (antibiotic inactivation)	Acetylation of the 2′-amino residue of kasugamycin, which inactivates the antibiotic	Yoshii et al. (2012, 2015)
		Deletion of *opp* and *dpp* (two permeases)	Deletion (reduced permeability)	Opp and Dpp are two major peptide ATP-binding cassette transporter systems. When deleted, kasugamycin cannot enter the cell	Ge et al. (2018)
Streptomycin	Aminoglycosides	*strA-strB*	Gene acquisition (antibiotic modification)	*strA-strB* encode phosphostranferases (aph(3″)-Ib and aph(6)-Id respectively) that modify streptomycin into a non-toxic form	Chiou and Jones (1993, 1995a), McManus et al. (2002), and Förster et al. (2015)
		*rpsL*	Point mutation (modification of the antibiotic targets)	*rpsL* encodes the ribosomal protein S12. Point mutations occur at codon 43 or rarely at codon 88 or 128, which prevent streptomycin to bind the ribosome	Chiou and Jones (1995b), Barnard et al. (2010), Zhang et al. (2011), Förster et al. (2015), and Escursell et al. (2021)
		*aadA1, aadA2*	Gene acquisition (antibiotic modification)	*aadA* genes encode aminoglycoside adenylyltransferases inactivating streptomycin	Schnabel and Jones (1999) and Xu et al. (2013)
Zhongshengmycin	Aminoglycosides	NA	NA	Increasing fatty acid biosynthesis	Wang Q. et al. (2021)
Bismerthiazol	Thiadiazol (inhibitor of histidine utilization pathway and quorum sensing)	NA	NA	NA	Zhu et al. (2013) and Liang et al. (2018)
Oxolinic acid	Quinolones (inhibitors of nucleic acids synthesis)	GyrA83 mutation	Point mutation (modification of the antibiotic targets)	*gyrA* encodes the DNA gyrase subunit A. The exact mechanisms are not defined yet	Maeda et al. (2007b)
Oxytetracycline	Tetracyclines (protein synthesis inhibitors)	*tetC*	Gene acquisition (active elimination)	Efflux of the antibiotic through a pump	Herbert et al. (2022)
Shenqinmycin	Heterocyclic antibiotic (phenazine) secreted by *Pseudomonas* spp. (accumulation of reactive oxygen species)	NA	Probably point mutation	Reduction of reactive oxygen species (ROS) production and/or increasing ability to metabolize ROS. The exact mechanisms are not defined yet	Pan et al. (2018)

NA, information not available.

### 3.1. Streptomycin resistance, mainly mediated by Tn*5393*

Streptomycin is used in plant protection against various PPB since the 1950s. The most common target of streptomycin is *Erwinia amylovora*, the causal agent of fire blight, which infects apple and pear trees, targeting leaves, flowers and shoots. Streptomycin is usually sprayed during bloom (McManus et al., 2002; Sundin and Wang, 2018).

Several mechanisms can be responsible for streptomycin resistance in PPB: (i) a point mutation of the *rpsL* gene, or (ii) the acquisition of *strA-strB* genes or *aadA* genes (Table 2). Other mechanisms have been suggested but they have not yet been confirmed and characterized. In *E. amylovora*, 95% of a total of 107 strains isolated in Mexico showed a mutation at codon 43 in *rpsL*, but the other 5% did not show *rpsL* mutation and did not carry *strA-strB* or *aadA* genes. The unknown resistance mechanism was not further investigated (de León Door et al., 2013). In *Clavibacter michiganensis*, another potential mechanism for streptomycin resistance was also suggested but needs further investigation (Lyu et al., 2019).

The *rpsL* gene encodes the ribosomal protein S12. A point mutation at codon 43 (mainly changing lysine (Lys) to arginine (Arg), rarely Lys to threonine or Lys to asparagine) prevents the binding of streptomycin to the ribosome. This mutation might be the most prevalent one since it retains a high environmental fitness even in the absence of streptomycin. Another less common mutation has been described at codon 88 (changing Lys to Arg). Both point mutations have been observed in various PPB (e.g., *E. amylovora*, *C. michiganensis* subsp. *michiganensis*, or *in vitro* in *Xanthomonas oryzae* pv. *oryzicola* or *Erwinia carotovora*, now named *Pectobaterium carotovorum*; Chiou and Jones, 1995b; Barnard et al., 2010; Zhang et al., 2011; Valenzuela et al., 2019; Escursell et al., 2021). In one streptomycin resistant strain of *C. michiganensis*, a point mutation at the 128th nucleotide of *rpsL* (Lys to Arg) was responsible for the resistance (Lyu et al., 2019). The *rpsL* chromosomal mutations might be the less worrying resistance mechanism because it is not prone to HGT.

Contrary to *rpsL* mutations, the acquisition of *aadA* or *strA-strB* genes requires more attention because of their presence on MGEs. Two alleles of *aadA* have been described, *aadA1*, the most prevalent allele, and *aadA2*. The *aadA1* gene was found in isolates of *X. oryzae* pv. *oryzae*, whereas *aadA2* conferred streptomycin resistance in a *Pseudomonas* strain (Schnabel and Jones, 1999; Xu et al., 2013). In *X. oryzae* pv. *oryzae*, three integrons carrying *aadA1* have been described, which was the first report of resistance integrons in PPB (see sections 3.5 and 3.6; Xu et al., 2013).

**Figure 2:**
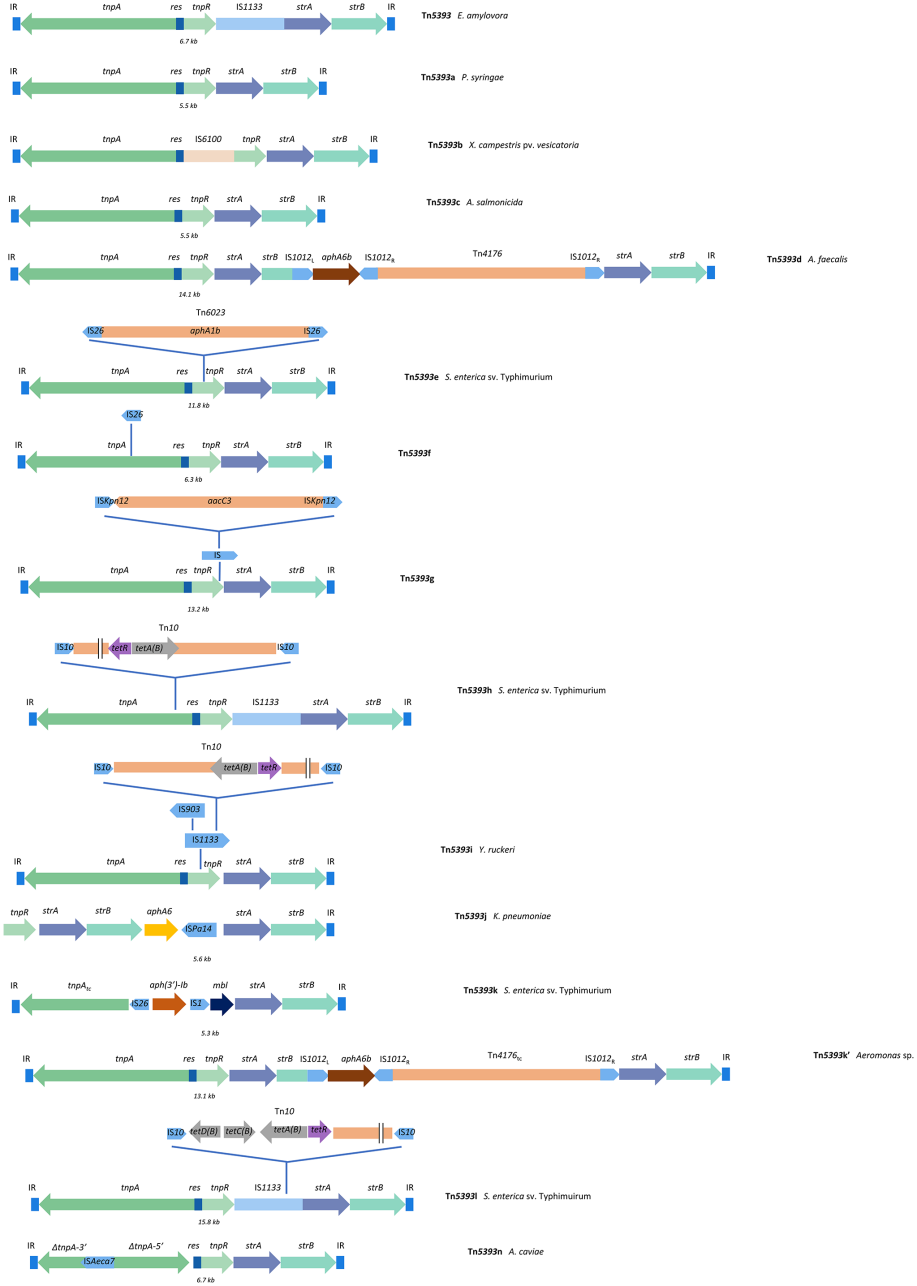
Schematic representations of the different variants of Tn*5393*, based on GenBank accession numbers and references reported in Table 3. *E. amylovora, P. syringae* and *X. campestris* pv. *vesicatoria* are PPB, while *Aeromonas* sp., *A. faecalis, S. enterica, Yersinia ruckeri* and *K. pneumoniae* are human pathogenic and/or environmental bacteria. Tn*5393* was first identified in *E. amylovora* and the successive variants discovered were then named alphabetically. It is however possible that Tn*5393*c, devoid of internal IS elements, compared to Tn*5393*, is the original version (Mindlin and Petrova, 2017). In this review, the original names of the different variants are used, but some publications have designated Tn*5393*c as Tn*5393* and Tn*5393*a as an IS*1133*-bearing variant. Also note than Tn*5393k* was described twice but the two versions were not identical and are distinguished here as Tn*5393k* and Tn*5393k*’. Genes are not drawn to scale. Tc: truncated.

The key actors in streptomycin resistance in PPB are the *strA-strB* genes, mainly found associated with Tn*5393*. This 6.7-kb transposon belongs to the Tn*3* family. In its most simple forms, it encodes a putative transposase (TnpA) and a resolvase (TnpR), followed by a putative recombination site (res), an insertion sequence (IS) element (IS*1133* in *E. amylovora*, IS*6100* in *Xanthomonas campestris*), and by the *strA-strB* genes (Figure 2). These two genes are commonly encountered in human pathogens and are responsible for many infections associated with streptomycin resistant bacteria (Chiou and Jones, 1993; Sundin and Bender, 1995; Förster et al., 2015). They are widely disseminated among Gram-negative bacterial pathogens and commensals from humans, probably because of streptomycin use in clinical contexts. The *strA-strB* genes are usually found on small plasmids, such as RSF1010 in human pathogens, while they are most commonly encoded on large conjugative plasmids in plant pathogens (Förster et al., 2015).

The *strA-strB* genes were identified in *E. amylovora* on plasmid pEA8.7, a plasmid very similar to the broad-host-range plasmid RSF1010, a 8.7-kb IncQ plasmid involved in streptomycin and sulfonamide resistances in bacterial human infections. It is non-conjugative but can be transferred horizontally through mobilization. Another RSF1010-like plasmid has also been observed in *Erwinia herbicola* in New Zealand, suggesting that IncQ plasmids of the RSF1010 family could be more widespread than previously thought (Palmer et al., 1997). It is hypothesized that the *strA-strB* sequence on RSF1010 originated from the insertion of Tn*5393* into the ancestral plasmid from which RSF1010 evolved. Indeed, the right inverted repeat of Tn*5393* (located downstream of *strB*) is conserved downstream of *strB* in RSF1010. This IR sequence would be the only vestige of Tn*5393* on RSF1010. The *strA-strB* genes carried by RSF1010 diverged over time from the intact *strA-strB* genes encoded on Tn*5393* from plant pathogens (Sundin, 2000).

Tn*5393* is found on a variety of plasmids (Table 3). It was initially discovered on the large conjugative plasmid pEa34 (34 kb) in *E. amylovora* (Chiou, 1991; Chiou and Jones, 1993). The spread of streptomycin resistance genes among *E. amylovora* or other bacteria (pathogenic or not) could thus be facilitated by this conjugative plasmid via HGT. Tn*5393* was also found on plasmid pEa29 from *E. amylovora*, where it can insert at several locations. This 29-kb plasmid is, unlike pEa34, unable to self-transfer to other strains, and is not related to pEa34. pEa29 is highly stable in *E. amylovora* and thus the integration of Tn*5393* suggests the stable establishment of the resistance in *E. amylovora* populations.

**Table 3:** Organisms, main plasmid host and mobility, associated with the different variants of Tn*5393* (Tn*5393*a to Tn*5393*l and Tn*5393*n).

Organism	Tn*5393* variant	ARGs carried by the transposon	Other MGEs present in the transposon	Plasmid; mobility; other ARGs carried by the plasmid	GenBank acc. Number	Reference(s)
*E. amylovora*	Tn*5393*	*strA-strB*	IS*1133*	pEa34; conjugative; none	M96392, M95402	Chiou (1991), Chiou and Jones (1993), and McManus et al. (2002)
	Tn*5393*	*strA-strB*	IS*1133*	pEa29; non-conjugative; none	NA	McManus et al. (2002)
	Tn*5393*a	*strA-strB*	None	pEU30; conjugative; none	NA	Foster et al. (2004) and Förster et al. (2015)
*P. syringae*	Tn*5393*a	*strA-strB*	None	pPSR1; conjugative; none	AY342395	Foster et al. (2004) and Sundin et al. (2004)
*X. campestris* pv. *vesicatoria*	Tn*5393*b	*strA-strB*	IS*6100*	pBV5-4a; ND; none	U20588	Sundin and Bender (1995) and Sundin (2002)
*A. salmonicida*	Tn*5393*c	*strA-strB*	None	pRAS2; conjugative; *sul2*, *tetA(31)* and *tetR(31)*	AF262622	L’Abée-Lund and Sørum (2000)
*A. faecalis*	Tn*5393*d	*strA-strB*, *aphA6b*, *blaPER-1*	IS*1012*_L_ and Tn*4176* including IS*1012*_R_, IS*1387*, IS*1066*, IS*Pa12*, IS*Pa13*, IS*Ppu17*	pFL424; non-conjugative; none	AJ627643	Mantengoli and Rossolini (2005)
*S. enterica* sv. Typhimurium	Tn*5393*a	*strA-strB*	None	pSRC27-I (I1 type plasmid); conjugative; various (e.g., *bla_CMY-2_*, *aadA*, *aac3*, *sul1*, *tetB*, *tetAR*, *blaTEM-1*, *aphA*)	CP058811	Harmer (2021)
	Tn*5393*e	*strA-strB*, *aphA1b*	Tn*6023* including IS*26*	pSRC125; ND; *sul1*, *dfrA5*, *tetA(B)*	GU562437	Cain and Hall (2011)
	Tn*5393*k	*strA-strB*, *aph(3′)-Ib*	IS*26*, IS*1*	pSW39; conjugative; *aadA2*, *aph(3′)-Ia*, *aac(6′)-Ib-cr*, *bla_DHA-1_*, *bla_OXA-1_*, *arr-3*, *tetA*, *floR*, *catB4*, *qnrB4*, *aac(6′)-Ib-cr*, *sul1*, *sfrA12*, *mphA*	NA	Yao et al. (2021)
	Tn*5393*l	*strA-strB*, *tetD(B), tetC(B), tetA(B), tetR(B)*	IS*1133*, Tn*10* including IS*10*	R64; conjugative; none	AP005147	Zhang et al. (2019)
Unidentified soil bacteria	Tn*5393*f	*strA-strB*	IS*26*	pHH1107; conjugative; gene homologous to *tetX*, *sul2*	FJ012881	Heuer et al. (2009), and Cain and Hall (2011)
Unidentified soil bacteria	Tn*5393*g	*strA-strB*, *aacC3*	IS*Kpn12*	pHHV35; conjugative; *aacC2*, *sul2*	FJ012882	Heuer et al. (2009) and Cain and Hall (2011)
*S. enterica* sv. Kentucky	Tn*5393*h	*strA-strB*, *tetA(B) and tetR*	IS*1133* and Tn*10* including IS*10*	pCVM29188_146; conjugative; none	CP001122	Fricke et al. (2009) and Cain and Hall (2011)
*Yersinia ruckeri*	Tn*5393*i	*strA-strB*, *tetA(B) and tetR*	IS*1133* including Tn*10*, IS*10* and IS*903*	pYR1; probably conjugative (carries genes essential for conjugative transfer); *sul2*, *dhfrl*	CP000602	Welch et al. (2007) and Cain and Hall (2011)
*K. pneumoniae*	Tn*5393*j	*strA-strB*, *aphA6*	IS*Pa14*	pJEG011; conjugative; *bla_OXA-48_*, *bla_CTX-M-14_*	KC354801	Espedido et al. (2013)
*Aeromonas* sp.	Tn*5393*k’	*strA-strB*, *aph(6)-Id*, *aph(3′)-VIb*, *aph(3″)-Ib*, *blaPER-1*	IS*1012*_L_ and Tn*4176* including IS*1012*_R_, IS*1387*, IS*1066*, IS*Pa12*, IS*Pa13*	ND	NA	Adamczuk and Dziewit (2017)
*A. caviae*	Tn*5393*n	*strA-strB*	IS*Aeca7*	Chromosomic	CP084031	Luo et al. (2022)

*E. amylovora, P. syringae* and *X. campestris* pv. *vesicatoria* are PPB, while *Aeromonas* sp., *A. faecalis, S. enterica, Yersinia ruckeri* and *K. pneumoniae* are human, animal, pathogenic or environmental bacteria. ND, not determined; NA, not available. Note that Tn5393k was described twice but the two versions were not identical and are distinguished here as *Tn5393k* and *Tn5393k’*. The genetic organization of these Tn5393 variants is illustrated in Figure 2.

Variants of Tn*5393* have been described (Figure 2; Table 3). Tn*5393*a was identified in several strains of *Pseudomonas syringae* and *Pseudomonas marginalis*, and lacks the IS*1133* of Tn*5393* (Sundin and Bender, 1993, 1995; Han et al., 2004). It was also detected in isolates of *E. amylovora*, located on the conjugative plasmid pEU30 (30 kb; Foster et al., 2004; Förster et al., 2015). Interestingly, the conjugative machinery used by pEU30 most resembles the VirB system from pPSR1 of *P. syringae* (Foster et al., 2004). It could be hypothesized that the Tn*5393*a element detected in *E. amylovora* originated from *P. syringae*. Tn*5393*b was identified in *X. campestris* and contains IS*6100* instead of IS*1133*. The presence of an insertion element increases the level of resistance in comparison to Tn*5393*a, which contains none (Sundin and Bender, 1995). IS*6100* in Tn*5393* from *X. campestris* is 100% identical to an element found in *Mycobacterium fortuitum*, *Pseudomonas aeruginosa* and *Flavobacterium* sp., suggesting its presence outside of PPB (Sundin and Bender, 1995). Also, the *tnpR* and *res* of Tn*5393*, Tn5*393*a and Tn*5393*b from *E. amylovora*, *P. syringae* and *X. campestris*, respectively, are identical (Sundin and Bender, 1995).

Other Tn*5393* variants have been found in bacteria not pathogenic to plants (Figure 2; Table 3). Tn*5393*c, reported in the fish pathogen *Aeromonas salmonicida* (L’Abée-Lund and Sørum, 2000), does not carry any insertion sequence, like Tn*5393*a, and sequence comparison revealed that Tn*5393*c and Tn*5393*a are essentially the same. The presence of Tn*5393*c in *A. salmonicida* is surprising because streptomycin is not used to control *A. salmonicida* in Norway, where the transposon was first described in this bacterium. However, huge amounts of other antibiotics (including oxytetracycline and sulfonamides) have been used and could have selected for the transposon located on pRAS2 which also carries oxytetracycline and sulfonamide resistance determinants (L’Abée-Lund and Sørum, 2000).

Tn*5393*d was identified in a clinical strain of *Alcaligenes faecalis*, which can be responsible for human infections related to contamination of the medical equipment (Mantengoli and Rossolini, 2005). Tn*5393*d carries additional antibiotic resistance genes and is found on pFL424, a 44 -kb non-conjugative plasmid. This transposon seems to have originated from the consecutive insertion of two composite transposons containing *aphA6b* (conferring resistance to kanamycin, streptomycin and amikacin) and *blaPER-1* (PER-1 extended spectrum beta-lactamase) genes (Mantengoli and Rossolini, 2005).

Tn*5393*e is a variant containing another transposon, Tn*6023*, and was found on the IncHI2 plasmid pSRC125. It was recovered from a multi-resistant *Salmonella enterica* serovar Typhimurium isolate of bovine origin. Tn*6023* itself contains the *aphA1b* gene, conferring resistance to various aminoglycosides. Surprisingly, strains containing this transposon did not show resistance to streptomycin, even though the *strA-strB* genes present on the element did not carry any inactivating mutations, suggesting that these genes were not expressed (Cain and Hall, 2011). Tn*5393*f and Tn*5393*g were discovered in unidentified soil bacteria where manure had been applied. Both transposons were found on low GC conjugative plasmids carrying other ARGs such as *sul2* or *aac3*, and *Acinetobacter* sp. was identified as the putative host (Heuer et al., 2009; Cain and Hall, 2011).

Tn*5393*h is derived from Tn*5393*a and includes Tn*10*, a tetracycline resistance transposon, inserted in the *tnpA* gene, which likely annihilates its mobility. Tn*5393*i contains IS*1133*, but this insertion element is not at the same location as in Tn*5393*a (namely in *tnpR*) (Cain and Hall, 2011). A variety of Tn*5393* variants sequences are also available in GenBank (Cain and Hall, 2011). Tn*5393*j from *Klebsiella pneumoniae* carries another aminoglycoside resistance gene, *aphA6*, but its *tnpR* was not functional and its *tnpA* gene is missing (Espedido et al., 2013). Two variants were named Tn*5393*k but, in fact, they are not identical; for the sake of clarity, they are referred to as Tn*5393*k and Tn*5393*k’ (Figure 2; Table 3). Tn*5393*k is similar to Tn*5393*c and Tn*5393*f, and carries both IS*26* and IS*1.* It is found in *S. enterica* sv. Typhimurium. The terminal inverted repeats (IRs) of Tn*5393*k exhibit 100% sequence identity with those in Tn*5393*, Tn*5393*c and Tn*5393*f (Yao et al., 2021). Tn*5393*k’ on the other hand is extremely similar to Tn*5393*d; the only difference is that it is lacking the IS*Ppu17* element (Adamczuk and Dziewit, 2017). Tn*5393*l carries the tetracycline resistance genes located in Tn*10* and is also found in *S. enterica* sv. Typhimurium (Zhang et al., 2019). Finally, Tn*5393*n was found in *Aeromonas caviae* (Luo et al., 2022).

Tn*5393* was also found in *Corynebacterium striatum*, *Campylobacter jejuni*, *P. aeruginosa* and *Snodgrassella alvi* (a honeybee gut symbiont; Sundin et al., 1995; Sundin, 2000; Ludvigsen et al., 2018). Similarly, Tn*5393*a was recently reported on a IncI1 type conjugative plasmid from *S. enterica* (Harmer, 2021) while Tn*5393*b was also found on the R64 plasmid of *S. enterica* sv. Typhimurium (Cain and Hall, 2011). Our recent BLAST run carried out on the Tn*5393* sequence revealed its presence in many other bacteria, human pathogens or not. Among others, Tn*5393* was found in *Citrobacter koseri*, *Corynebacterium crudilactis*, *Enterobacter cancerogenus*, *Enterobacter cloacae*, *Enterobacter hormaechei*, *Escherichia coli*, *K. pneumoniae*, *Klebsiella quasipneumoniae*, *Klebsiella variicola* and *S. enterica*, as well as in the plant root-associated bacterium *Pseudomonas putida*. However, this does not demonstrate that direct transfer of Tn*5393* occurred between PPB and human pathogens but it certainly indicates that Tn*5393* is much more widespread than previously thought. Similarly, the fact that this transposon was detected in diverse bacteria (Gram-negative as well as Gram-positive) in distinct geographical locations shows that it might be accessible to a large range of organisms.

The sequence of Tn*5393*a also retrieved numerous matches in the BLAST analysis, mostly in antibiotic resistant human pathogens, and in two PPB, *P. syringae* pv. *actinidiae* (plasmid pMG2_SR198) and *Agrobacterium tumefaciens* (plasmids pTi and pAt). Finally, Tn*5393*d displays strong similarities with plasmid p17-84_OXA of *Acinetobacter baumannii*, a critical multi-resistant pathogen in human health for which new antibiotics are needed according to the WHO (World Health Organization, 2017), and with pOXA58_100004 from *Acinetobacter pitii*. Tn*5393*e was found in other human bacteria, among which some are pathogenic.

In relation with human health implications, several important findings are worth mentioning. First, a tigecycline resistance mechanism called “resistance-nodulation-division” (RND) family efflux pump (*tmexCD1-toprJ1* pump) was identified in *K. pneumoniae* isolates from humans and chickens. The corresponding gene *tmexCD1-toprJ1* appeared to be found in Tn*5393* and the authors suggest that it originated from the chromosome of *Aeromonas* spp. through Tn*5393*-mediated translocation (Sun et al., 2020). Second, a tigecycline resistance gene, *tet(Y)*, was identified on a plasmid of a multi-resistant clinical isolate of *A. baumannii*. This plasmid carried numerous ARGs, including *strA*, *strB*, *aac(6′)-Ib3*, *msr(E)*, *mph(E)*, *floR*, *ARR-3*, *sul1*, *dfrA19*, *tet(39)* and *tet(Y)*. Interestingly, the *tet(Y)* gene was located inside the sequence of Tn*5393* and the authors also concluded that Tn*5393* played a role in its transmission from *Aeromonas* spp. (Wang Z. et al., 2021). Lastly, in Australia, a multi-resistant *A. baumannii* strain that contains a fragment of Tn*5393*, was isolated, showing again its potential of ARGs spreading (Post and Hall, 2009). It is interesting to note that some species of *Aeromonas* or *Acinetobacter* can also be found in the environment and genetic transfers in these genera should be carefully considered.

Recently, the genomic evolution of Tn*5393* and kin was examined and different steps were proposed on how Tn*5393* could have acquired various MGEs and became more complex.^1^ It is also suggested that other variants of Tn*5393* could exist (Lima-Mendez et al., 2020; Ross et al., 2021). Additionally, when comparing the different TnpA sequences from Tn*5393* and its variants, it is rather complicated to establish their exact filiation and to determine how exchanges between bacterial species have shaped their structures. Still, the complexity of Tn*5393* is apparently greater among human or animal pathogens than in PPB (Figure 2; Table 3).

### 3.2. Oxytetracycline resistance

Oxytetracycline belongs to the tetracycline class of antibiotics and is the second most used antibiotic in plant agriculture, mainly against fire blight (*E. amylovora*) in the case of streptomycin resistance reports. However, it is only used as a second line of defense because it is considered as less effective than streptomycin (McManus et al., 2002; Sundin and Wang, 2018). Various genes can be involved in tetracycline resistance: (i) expression of efflux pumps, (ii) enzymatic inactivation of the antibiotic or (iii) ribosomal protein protecting from the action of tetracyclines (Chopra and Roberts, 2001).

Concerning PPB, cases of oxytetracycline resistance are rarely reported. To the best of our knowledge, oxytetracycline resistance has not been observed yet in field isolates of *E. amylovora*, although, resistant strains can be obtained *in vitro*. In fact, the RP1 plasmid carrying an oxytetracycline resistance gene can be transferred from both *E. herbicola* and *P. syringae* pv. *syringae* to *E. amylovora.* Nevertheless, after pathogenesis, the plasmid phenotype could only be recovered in 0.03% of the isolates, suggesting that it may not be stable in that species (Lacy et al., 1984).

Oxytetracycline resistance cases have been reported in *P. syringae* (da Silva and Lopes, 1995; Spotts and Cervantes, 1995; Hwang et al., 2005), *Xanthomonas arboricola* pv. *pruni* (Herbert et al., 2022) and *A. tumefaciens* (Luo and Farrand, 1999). The resistance mechanisms were not described, except in *X. arboricola* pv. *pruni*, where it was shown that the tetracycline resistance gene *tetC* was carried by a plasmid, coding for an efflux pump (Herbert et al., 2022). Given the plethora of known tetracycline resistance genes (36 efflux pump genes, 13 ribosomal protection protein genes, 13 enzyme genes, 1 other gene conferring an unknown mechanism of resistance and 11 mosaic genes reported so far; Chopra and Roberts, 2001; Van Hoek et al., 2011; Roberts, 2021), others could be present in PPB but they have not been described yet. When searching for identity while running BLAST on PPB genomes with the sequence of *tetC* mentioned above, several strains present sequences with high identity percentage, such as *X. arboricola* pv. *pruni*, *Burkholderia cepacia*, *Agrobacterium fabrum* and several *Streptomyces*, which could be an indication that tetracycline resistance genes are present in other PPB.

### 3.3. Kasugamycin resistance

Kasugamycin is an aminoglycoside antibiotic used only in plants, not in human nor veterinary medicine. In *E. coli*, point mutations in various genes (*ksgA*, *ksgB*, *ksgC* and *ksgD*) were shown to confer resistance (Sparling, 1970; Sparling et al., 1973; Yoshikawa et al., 1975; Fouts and Barbour, 1981; Yoshii et al., 2012). In PPB, different mechanisms have been identified (Table 2). The presence of *aac(2′)-IIa* gene in *Burkholderia glumae* and *Acidovorax avenae* ssp. *avenae* was shown to cause the resistance to kasugamycin (Yoshii et al., 2012). The Aac(2′)-IIa acyltransferase inactivates kasugamycin via acetylation of the 2′-amino residue of the antibiotic. In the chromosome of *B. glumae*, this acetyltransferase is encoded on the IncP genomic island, suggesting that this kasugamycin resistance gene might have been acquired by HGT and that the gene could hypothetically spread among PPB (Yoshii et al., 2012). The *aac(2′)-IIa* gene was later found on a conjugative IncP-1β plasmid, pAAA83, in an *A. avenae* ssp. a*venae* strain, raising even more concern about the potential transmission of this resistance gene (Yoshii et al., 2015). In *E. amylovora*, resistance to kasugamycin and blasticidin S is induced by the *in vitro* deletion of both permease genes *opp* and *dpp*, suggesting that Opp and Dpp act synergistically to transport these antibiotics. This deletion has not been observed in field isolates, so the relevance of such mechanism in the environment remains to be assessed (Ge et al., 2018). Similarly, *in vitro* mutation of the *ksgA* gene in *E. amylovora* confers kasugamycin resistance but it requires a two-step mutational process and these mutants displayed a considerably reduced fitness (McGhee and Sundin, 2011; Ge et al., 2018).

### 3.4. Oxolinic acid resistance

In PPB, the exact mechanisms involved in OA resistance have not been described in great details (Table 2). *In vitro* mutants of *B. glumae* resistant to the OA quinolone were obtained from strains isolated in rice fields in Japan, where the antibiotic is used as PPP. GyrA83 mutation (substitution of serine by arginine or isoleucine at position 83) seemed to be involved in the resistance mechanism (Maeda et al., 2007a,b). Others have also demonstrated that OA resistant *B. glumae* could be obtained *in vitro*, however such mutants were unable to survive in paddy fields (Hikichi et al., 1998, 2001).

In Israel, OA was introduced in 1997 when the use of streptomycin was abandoned. About 2 years later, OA resistant *E. amylovora* and *B. glumae* were reported. Yet, the exact mechanisms leading to OA resistance in these strains remained unknown, although it was suggested that it might consist into chromosomal mutations rather than gene acquisition through HGT (Manulis et al., 2003; Kleitman et al., 2005).

### 3.5. Gentamicin resistance

Resistance to the gentamicin aminoglycoside was only reported in *X. oryzae* pv. *oryzae*, where the acquisition of the integron-borne *aacA3* gene was incriminated. As indicated in Table 2, *aacA3* encodes an aminoglycoside-3’-*N*-acetyltransferase enzyme that deactivates gentamicin (Xu et al., 2013).

### 3.6. Co-occurrence of antibiotic resistance genes, cross-resistance and resistance to other antibiotics in plant pathogenic bacteria

The *aadA1* gene was found in *X. oryzae* pv. *oryzae* on three integrons and associated with other antibiotic resistance gene cassettes such as *aacA3* (conferring resistance to tobramycin, kanamycin, gentamicin and netilmicin) or *arr3* (conferring resistance to rifampicin; Xu et al., 2013). Both *aadA1* and *aadA2* also confer spectinomycin resistance. The co-occurrence of streptomycin and gentamicin resistance is therefore possible and it is then reasonable to speculate that these gene cassettes could be co-transferred under the selective pressure of streptomycin or gentamicin.

In a recent study conducted in South Carolina (United States), strains of *X. arboricola* pv. *pruni* were isolated and characterized for their resistance to both streptomycin and oxytetracycline. They carried a plasmid encoding *tetC*, *tetR* and *strA-strB*, with a region similar to Tn*5393* (Herbert et al., 2022). It was previously described that, when isolating epiphytic bacteria (not PPB) from Michigan apple orchards, almost all tetracycline resistant strains were also streptomycin resistant and that tetracycline resistance genes were found on plasmids that also carried Tn*5393* (Schnabel and Jones, 1999). Similarly, several plasmid-borne tetracycline resistance elements have been found in various epiphytic bacteria and usually the tetracycline resistance was associated with transposons, mainly Tn*5393* (carried by an uncharacterized plasmid; Schnabel and Jones, 1999). These data indicate that PPB could become resistant to different antibiotics through the acquisition of resistance genes from other plant-associated bacteria if the genes are present on MGEs.

In 2005, several *P. syringae* were isolated and assessed for their antibiotic resistance to six antibiotics. One strain of *P. syringae* pv. *syringae* was resistant to kanamycin and tetracycline. Eight isolates were resistant to streptomycin, 16 isolates were resistant to rifampicin and 36 to chloramphenicol. Fifty-five strains in total were resistant to ampicillin. Some of these strains were resistant to several antibiotics, and although the resistance mechanisms were not characterized, this study further highlights the possibility of ARGs co-occurrence (Hwang et al., 2005).

Along the same lines, an OA resistant *B. glumae* was reported to display cross-resistance with other quinolones such as ciprofloxacin (Hikichi et al., 2001), while another isolated *B. glumae* was resistant to polymyxin B (Paz-Carrasco et al., 2018).

Finally, it is worth mentioning three studies that have addressed resistance to antibiotics mainly used in China. First, it was possible to obtain *X. oryzae* pv. *oryzae* strains resistant to phenazine-1-carboxylic acid (PCA), also called shenqinmycin in China, after *in vitro* exposition to increasing concentrations of the antibiotic (Pan et al., 2018). Second, zhongshengmycin resistant strains of *X. oryzae* were obtained *in vitro* and the resistance was related to increasing fatty acid biosynthesis. The exact underlying mechanisms are not known yet (Wang Q. et al., 2021). Third, bismerthiazol resistant mutants of *X. oryzae* pv. *oryzae* could also be induced, both *in vivo* and *in vitro* (Zhu et al., 2013). However, these mutants were all obtained *in vitro* or induced, so the natural occurrence of such resistance remains to be demonstrated. Nevertheless, this indicates that *X. oryzae* pv. *oryzae* is highly adaptable and may easily develop resistances.

## 4. Link between the use of antibiotics and the development of resistance in plant pathogenic bacteria and other plant-associated bacteria

This section mostly focuses on streptomycin, which has been used the longest in plant agriculture, compared to other antibiotics, and remains the most used antibiotic in this context today. Scientific evidence that can directly link the use of streptomycin and the abundance of streptomycin resistance genes or streptomycin resistant strains are relatively scarce. In fact, although several studies suggest that the application of streptomycin in fields does not influence the abundance of resistance genes in PPB and other surrounding bacteria, opposite conclusions have been reached by other studies.

### 4.1. Effects on the bacterial diversity and on antibiotic resistant bacteria

Several studies reported that the application of streptomycin on orchards did not, or not adversely, affect the bacterial population of the soil (Walsh et al., 2013; Shade et al., 2013a) or described minimal alterations with a slight decrease in phylogenetic diversity of bacterial communities of apple tree flowers (Shade et al., 2013b), on the short-term. Another study indicated a decrease of diversity in streptomycin-treated soil (Tolba et al., 2002). When looking at the bacterial communities of apple leaves, a higher frequency of streptomycin resistant bacteria was observed in samples that were not treated with streptomycin (Yashiro and McManus, 2012). However, they only focused on apple leaves and not on the other bacterial communities (soil or roots) that could be affected differently. This work also dealt with the overall bacterial community, and not with specific species that could become resistant without significantly increasing the overall abundance of resistance. Moreover, as indicated before, streptomycin application could select for other ARGs.

The effects of streptomycin and kasugamycin application on bacteria in the apple phyllosphere have been investigated. While the use of streptomycin did not result in any increase of streptomycin resistant *E. amylovora*, it was correlated with an increase in resistance in other epiphytic bacteria, such as *Pantoea agglomerans* or *Pseudomonas* spp. No kasugamycin resistant bacteria could be isolated, however the antibiotic application changed the microbial spectrum in the orchard (Tancos and Cox, 2017). Along the same lines, there was no influence on the abundance of culturable oxytetracycline and gentamicin resistant bacteria when these antibiotics where applied, as compared to untreated soils (Rodríguez-Sánchez et al., 2008). On the opposite, kasugamycin resistant Gram-negative bacteria (mainly residents of the plant phyllosphere or colonists of apple, such as *P. agglomerans*, *Pseudomonas graminis*, *P. syringae* and *Stenotrophomonas* spp.) could be isolated in another study from orchard soil, apple flowers and leaves treated with kasugamycin (McGhee and Sundin, 2011).

Sundin et al. (1995) observed the distribution of streptomycin resistance transposon Tn*5393* in the apple phylloplane and soil of ornamental pear (that had received previous applications of streptomycin) and tomato (no prior exposure to streptomycin; Sundin et al., 1995). The recovery of streptomycin resistant bacteria was generally higher in soil samples, but the highest occurrence was from phylloplane samples from an orchard where streptomycin had been applied. They showed that the use of streptomycin for plant protection can also select for streptomycin resistance in non-target commensal bacteria that inhabit either plant surfaces or the surrounding soil (Chiou and Jones, 1993; Sundin et al., 1995). They investigated the risk of co-selection of other antibiotic resistance genes (tetracycline), but found no evidence that the use of streptomycin increased tetracycline resistance (Sundin et al., 1995). One should however note that streptomycin and tetracycline resistance genes were recently found on the same plasmid (Herbert et al., 2022).

In a bioinformatic analysis, where a total of 127 genomes of *E. amylovora* from different geographic regions were examined, the greatest number of streptomycin resistant isolates was observed in Western North America (in particular in British Columbia, Canada), where streptomycin was used, at least during the survey (1993–1998; Sholberg et al., 2001; Parcey et al., 2020). Several additional studies showed that the orchards where streptomycin use is the greatest were usually those where the highest numbers of streptomycin resistant bacteria were detected (Chiou, 1991; Norelli et al., 1991; Burr et al., 1993).

Regarding oxytetracycline resistance, the finding of a tetracycline resistant isolate of *A. tumefaciens* could not be linked to oxytetracycline application (Luo and Farrand, 1999). On the opposite, antibiotic sprays of streptomycin and oxytetracycline were positively correlated with resistance in *P. syringae* pv. *syringae* (Spotts and Cervantes, 1995). In epiphytic bacteria, the use of oxytetracycline generally resulted in less abundant bacterial populations than when streptomycin was used, but streptomycin resistant isolates were more common than the tetracycline resistant ones. Moreover, tetracycline resistant strains could be observed in orchards where tetracycline had not been applied, although higher numbers of tetracycline resistant bacteria could be detected in another orchard where oxytetracycline was applied (Schnabel and Jones, 1999).

In Israel, the introduction of OA to replace streptomycin led to the isolation of OA resistant *E. amylovora* and *B. glumae*. However, the incidence of OA resistance in *E. amylovora* was sporadic and irrespective of the number of sprays applied and the severity of the disease (Kleitman et al., 2005). In another study also conducted in Israel after introduction of OA, several OA resistant strains were isolated from different orchards but none was resistant to both streptomycin and OA (Manulis et al., 2003). Concerning gentamicin resistance, the only report available in *X. oryzae* pv. *oryzae* in China has not been linked to the use of gentamicin (Xu et al., 2013).

### 4.2. Effects on the occurrence of antibiotic resistance genes

The abundance of streptomycin and tetracycline resistance genes in flowers, leaves and soil samples from orchards treated with streptomycin in Switzerland was analyzed using multiplex qPCR (Duffy et al., 2014). Three orchards that had not been treated with streptomycin prior to the assays were used, and samples were collected over a three-year period. By using qPCR to assess the abundance of ARGs, this study overcame the intrinsic problem linked to culture-dependent assays where unculturable bacteria are overlooked. This method allows for a qualitative assessment of ARGs in the complete apple tree ecosystem but does not identify the species that contain the resistance genes. The results indicated no consistent increase in streptomycin resistance genes in streptomycin-treated samples. Tetracycline resistance genes were also quantified in this study, and no increase was reported with streptomycin use. It is however important to note that due to the nature of the technique used, streptomycin resistance genes, notably *strA-strB* and *aadA*, had to be selected for the screening. Other potential resistance mechanisms might have been ignored.

Similarly, the abundance of various tetracycline resistance genetic determinants and several gentamicin resistance genes was investigated under field conditions with five applications of oxytetracycline and gentamicin during 16 months. This study indicated that the occurrence of these genes was not related to the application of antibiotics (Rodríguez-Sánchez et al., 2008).

### 4.3. Resilience to antibiotic resistances

Little is known about the resilience of an ecosystem to antibiotic resistances, years after the discontinuation of the antibiotic use.

In Israel, streptomycin was removed from the list of approved antibiotics for plant agriculture in 1997 and has not been used since. A decline in the incidence of streptomycin resistance in *E. amylovora* was observed in the subsequent years (from 57% in 1998 to 15% in 2001). Surprisingly, within 4 years, the resistance had almost disappeared (Manulis et al., 2003).

Other studies have been conducted in a few American states. In California, streptomycin resistance among bacteria from orchards declined from 1973 to 1977. However, streptomycin resistant *E. amylovora* strains could still be isolated, even though streptomycin had not been applied in these orchards since 1971 (Schroth et al., 1979). In Washington state, streptomycin resistant *E. amylovora* strains could still be isolated 5 years after termination of streptomycin use (Loper et al., 1991). Schnabel and Jones (1999) showed that streptomycin resistance did not diminish at their study site over the course of 2 years in the absence of the selection pressure (Schnabel and Jones, 1999). In Michigan, a decline in the incidence of streptomycin resistance was observed from 1991 to 1992 when oxytetracycline was used rather than streptomycin. However, streptomycin resistance in *E. amylovora* quickly reoccurred in 1993 when the use of streptomycin was resumed (McManus and Jones, 1994). This could suggest that streptomycin resistant strains have lower fitness than streptomycin sensitive strains, although Loper et al. (1991) found streptomycin resistant strains where this antibiotic had never been applied, suggesting similar fitness capabilities under no selective pressure (Loper et al., 1991).

### 4.4. What about antibiotic resistance genes naturally present in soils?

It was shown that there is a high presence of streptomycin resistance in agricultural sites without streptomycin treatment history, and that HGT of *strA* and *strB* occurred regardless of soil treatment with antibiotics (Tolba et al., 2002). It has also been shown that *strA* gene is more prevalent in soils from compost, forest or agriculture samples than from vegetable gardens, apple orchards or mixed fruit orchards (Popowska et al., 2012). However, in the same study, *strB* was less prevalent in compost, forest or agricultural soils than in apple orchards or mixed fruit orchards. The *aadA* gene followed the same pattern as *strB* but was only detected in agricultural soil at very low rates, while absent in compost or forest soils. It was rather prevalent in vegetable gardens, apple orchards or mixed fruit orchards.

It is also worth mentioning that the streptomycin resistant transposon Tn*5393* was detected in bacteria with no prior exposure to streptomycin. It seems that this transposable element is indigenous to both phylloplane and soil microbial communities (Sundin et al., 1995). In fact, *strA-strB* and Tn*5393* are both present in non-target bacteria (Chiou and Jones, 1993; Sundin et al., 1995). Gentamicin resistance genes are also commonly found in environments associated with sewage effluent or farm animals (Heuer et al., 2002).

## 5. Discussion

There are many studies reporting the presence of antibiotic resistant bacteria and ARGs in orchards. This does not establish, *per se*, a link with the use of antibiotics. Research on the relationship between antibiotic use and antibiotic resistance in PPB is largely incomplete. Many other factors could drive the emergence of resistant strains, such as cross-resistance to other PPPs, e.g., fungicides or copper, the use of manure, irrigation water, sewage sludge or antibiotic resistance from animal or human uses. As of now, there are not enough data to support either view with confidence. More large-scale and long-term research in fields with non-treated controls are needed to be able to have statistically relevant data on the issue.

What is known for sure is that streptomycin resistance is mainly mediated by *strA-strB* found on the Tn*5393* transposon, which seems to be rather widespread. The *strA-strB* genes found in PPB are broadly disseminated in human pathogens, although the alleles of the genes are different, suggesting diverse routes of acquisition (Sundin and Bender, 1996; Sundin, 2000, 2002). Detection of similar *strA-strB* genes in PPB and in human pathogens does not automatically mean that transfer occurred directly between these organisms. In the clinical context, the main problem with Tn*5393* is its ability to translocate and mobilize other ARGs rather than streptomycin resistance *per se*. Strains of two major human pathogens (*S. enterica* and *K. pneumoniae*) have been found to carry Tn*5393* variants, as well as several species of *Aeromonas*, which could indicate that the transposon is also circulating in this bacterial genus. The selection of Tn*5393* in plant-associated bacteria driven by extensive streptomycin use in agriculture could create a reservoir that may negatively influence the antibiotic resistance crisis. The fact that the same Tn*5393* can be found in PPB and in *S. enterica* is a worrying fact.

By using antibiotics on plants, not only are the associated resistance genes selected, but also the MGEs that carry these ARGs, which is often underestimated. Tn*5393* is the only well-documented example, so far, of MGE associated with antibiotic resistance in PPB, but it cannot be excluded that other “mobile” resistance genes are yet to be discovered or to emerge. Besides, it is known that the use of antibiotics selects not only for the resistance genes, but also contributes to the evolution of complex vectors (MGEs) encoding several ARGs (O’Brien, 2002), which is a plausible phenomenon also in PPB.

The translocation of ARGs among plasmids thanks to transposons also potentially accelerates the spread of ARGs (Yao et al., 2022). The fact that Tn*5393* can translocate to other plasmids also suggests the risk of insertion into other conjugative plasmids, which could lead to further spread of the resistance genes (Falkenstein et al., 1989; McManus et al., 2002; Llop et al., 2006). This suggests Tn*5393* is evolving and that IS or other transposons have inserted into its structure resulting into more complex, better-fit elements, potentially carrying other ARGs. Tn*5393* structures are still relatively simple in PPB (Figure 2; Table 3), but in human or animal pathogens, variants of the transposon show the potential complexity that could be attained in PPB within a few years, with the formation of complex transposons simultaneously carrying several ARGs.

Regarding the other antibiotics used in plant protection, less information is yet available regarding the resistance risk that they entail. Since the beginning of its use in plant protection, there have been very few reports of tetracycline resistance in PPB. It could therefore be assumed that oxytetracycline resistance is not an issue on the short-term. However, risks related to the transfer of tetracycline resistance genes from epiphytic bacteria to human pathogens or PPB cannot be excluded. Besides, the uncertainty linked to the lack of studies cannot be underestimated. When tetracycline resistance is present, it is typically encoded on plasmids associated with Tn*5393*, which indicates a potential link between streptomycin and tetracycline resistance and the potential dissemination of both resistances at the same time (Schnabel and Jones, 1999; Herbert et al., 2022).

A better understanding of the extent to which epiphytic bacteria can serve as reservoirs of ARGs for PPB or human pathogens is crucial. In fact, it is very likely that *strA-strB* and Tn*5393* were first transferred from non-target bacteria to plant pathogens, resulting in streptomycin resistant PPB (Chiou and Jones, 1993; Sundin et al., 1995). Regarding the case of oxytetracycline resistance potential acquisition, the availability of tetracycline resistance genes in the bacterial populations exposed to tetracycline and their ability to transfer to *E. amylovora* are clear factors contributing to the risk of tetracycline resistance selection in this pathogen. Even though tetracycline resistance in *E. amylovora* by chromosomal mutation does not easily occur (Lacy et al., 1984), the use of tetracycline could potentially lead to the selection of resistant strains of *E. amylovora* or other PPB through the acquisition of tetracycline resistance genes from other plant (or soil) bacteria if the genes are present on MGEs (Chiou and Jones, 1993; Sundin et al., 1995).

Although presently kasugamycin resistance does not appear as a major problem in plant agriculture, the recent emergence of a transmissible kasugamycin resistance gene constitutes a considerable threat for the effective control of the diseases involved, especially because the resistance gene might spread to other bacteria through HGT via MGEs. Dissemination of kasugamycin resistance among PPB is therefore a possibility. Kasugamycin is often considered as an interesting alternative to the other antibiotics used in plant protection because of its non-use in human or veterinary medicine. In fact, it was shown to be efficient for the management of fire blight when streptomycin resistant strains were present (McGhee and Sundin, 2011). Conversely, in this same study, Enterobacteriaceae and *Pseudomonas* spp. resistant to both kasugamycin and streptomycin were isolated, which is concerning because of the potential resistance transfer to *E. amylovora* and the potential link between these two ARGs. The authors dismissed the potential cross-resistance among singly resistant spontaneous mutants for either kasugamycin or streptomycin, because kasugamycin resistant *E. amylovora* strains were sensitive to streptomycin and vice versa. However, this does not mean that the double resistance could not arise in the future. An increasing number of antibiotics restricted to plant protection are proposed to control bacterial diseases, but cross-resistances and selection of antibiotic resistance vectors should be investigated.

Nowadays, very little information is known about gentamicin resistance in PPB. However, the only gentamicin resistance report was due to a gene found on an integron, which may then be able to spread through HGT. Rare cases of resistance to OA have been observed in Israel in isolates of *E. amylovora* and *B. glumae*, where the antibiotic is used as PPP, but the exact resistance mechanisms were not characterized, though it seems that they only involved chromosomal mutations so far (Manulis et al., 2003; Kleitman et al., 2005), such as the GyrA83 mutation observed *in vitro* (Maeda et al., 2007b).

Because of the main way of application of antibiotics on plants (spraying), it was suggested that it might result in a limited selection for resistance, due to photodegradation, soil adsorption or deactivation and the substantial dilution (McManus, 2014). However, contrarily to animal and human medicine, where the use of antibiotics is rather controlled, when used in plant health, antibiotics are sprayed on a large scale and with relatively high doses, probably notably because of the factors cited above, which is also a source of great concerns due to the large environmental exposure. Besides, it is well-known that antibiotic concentrations already well below the minimal inhibitory concentrations (MICs) select for antibiotic resistant bacteria (Bengtsson-Palme and Larsson, 2016), both pathogenic and commensal, which can then become vectors of ARGs. It is also worth noting that, in this review, the purity of the antibiotic, the potential role of the excipients (Killiny et al., 2020), the way of use (spraying or injection) as well as the type of crops were not taken into consideration. Yet, they all play a role in the amount of antibiotic that is effectively applied and the potential subsequent selection pressure on bacteria.

Efficient and safe alternative control measures are urgently needed to manage bacterial diseases in plant health, to avoid resorting to antibiotics in the first place. Reducing the need to use antibiotics would be the safest way to avoid the selection and emergence of antibiotic resistance. Many innovative control measures are being explored as new potential alternatives to antibiotics, notably antagonistic bacteria or competitive fungi (Poveda et al., 2021; Poveda and Baptista, 2021), bacteriophages (Buttimer et al., 2017; Grace et al., 2021) or for the control of animal vectors of PPB (Di Serio et al., 2019; Vicente-Díez et al., 2021). In the European Union, despite the absence of authorization of antibiotic use, fire blight is not so much of a problem because other strategies are in place to control this bacterial disease. In other locations of the world, using antibiotics is often the choice of convenience, but this strategy also hampers the development and application of alternative methods.

## 6. Concluding remarks

Even though the use of antibiotics in plant protection is considered relatively low in comparison to the use for human and veterinary medicine, the impact it could potentially have on the phytobiome cannot be overlooked, with potential unintended side effects such as the development of antibiotic resistance. It must also be stressed that PPB are not the only bacteria and microorganisms associated with plants (e.g., fungi, that can also be controlled through antibiotics with antifungal properties, as well as soil bacteria, might also develop resistance). Another aspect not developed in this paper is the use of biocontrol agents to control PPB or fungi, that might themselves carry ARGs. They are also sometimes spread heavily on plants, and are currently approved in Europe and other countries, such as *Streptomyces lydicus* WYEC 108 [US Environmental Protection Agency Office of Pesticide Programs, 2005; European Food Safety Authority (EFSA), 2013; European Food Safety Authority (EFSA) et al., 2020]. They could also represent a source of ARGs and MGEs, potentially interacting with the microorganisms associated with plants.

The contribution of MGEs to the spread of antibiotic resistance in PPB, as well as their ability to transfer to other bacteria, need to be further investigated carefully. In fact, the only well-studied example of ARGs vector in PPB, Tn*5393*, is concerning because of its occurrence outside of PPB and its structure evolving into complex associations of MGEs and ARGs. The effect of antibiotic use on non-pathogenic plant-associated bacteria is not well studied and largely unknown, still they represent another piece of the puzzle that could allow MGEs to travel from crops to the environment and to the human consumers. Whether the use of antibiotics in plant health has an impact on the global problematic of antibiotic resistance remains unanswered.

The extent of the risk of resistance that goes along with antibiotic use in plant protection cannot be excluded given the current lack of data. To reduce the resistance risk, the adoption of strong antimicrobial stewardship practices (Miller et al., 2022) is essential. The development of surveillance programs practical and achievable also by low- and middle-income countries that are harmonized and collect quantitative data on the use and sales of antibiotics, as well as the crops and area of their application is necessary to better understand the situation and assess the risks of antibiotic resistance selection and spread. Accurate data on the amounts of antibiotic used in different crops are crucial to better identify and quantify the related risk of development of antibiotic resistance.

Unravelling the risks potentially associated with the use of antibiotics and potential gene transfers among PPB species via MGEs is urgent, which is why surveillance in plants and soils needs to be improved. In order to assess the potential spread of these ARGs, it is also of uttermost importance to develop and use new detection methods (modern tools to carry out genetic analyses). Whole genome sequencing (WGS) of resistant field strains and metagenomics of field samples could clarify where the genes are located and how they can be transferred (presence on MGEs), as well as contribute greatly to resistance prediction (Arango-Argoty et al., 2018). In addition, rapid and inexpensive tests and/or tools are needed to facilitate the identification of PPB and to characterize their resistomes.

## Author contributions

MV, TB, and CB carried out the original literature screening under the supervision of CB, JM, and M-PM-L. MV drafted the manuscript with inputs from JM and CB. MV, EL, GS, FS, M-PM-L, JM, and CB edited, revised and approved the final version of the manuscript. All authors contributed to the article and approved the submitted version.

## Funding

This work was supported by the European Food Safety Authority (EFSA, grant to MV and TB) under Agreement Number GP/EFSA/ALPHA/2020/02.

## Conflict of interest

The authors declare that the research was conducted in the absence of any commercial or financial relationships that could be construed as a potential conflict of interest.

## Publisher’s note

All claims expressed in this article are solely those of the authors and do not necessarily represent those of their affiliated organizations, or those of the publisher, the editors and the reviewers. Any product that may be evaluated in this article, or claim that may be made by its manufacturer, is not guaranteed or endorsed by the publisher.

## Author disclaimer

The authors EL, GS, and FS are employed by the European Food Safety Authority (EFSA). However, the present article is published under the sole responsibility of the authors and may not be considered as an EFSA scientific output. The positions and opinions presented in this article are those of the authors alone and do not necessarily represent the views/any official position or scientific works of EFSA. To know about the views or scientific outputs of EFSA, please consult its website “www.efsa.europa.eu”.

## Footnotes

^1^https://tncentral.ncc.unesp.br/TnPedia/index.php/Transposons_families/Tn3_family#The_Tn163_Clade
