# Supplementary material for: On the use of antibiotics to control plant pathogenic bacteria: a genetic and genomic perspective
Source: Front Microbiol. 2023 Jun 27;14:1221478. doi: 10.3389/fmicb.2023.1221478 (PMC10333595; doi:10.3389/fmicb.2023.1221478)
Supplement: Supplementary file 1 [file Table_1.docx]

**Supplementary materials**

**Table S1.** Examples of antibiotics tested as plant protection products (PPPs), as of January 2023. Information might not be exhaustive, given the large amount of agents tested as PPPs.

| Antibiotics tested as PPPs | | | | |
| --- | --- | --- | --- | --- |
|  | Antibiotic | Countries or region | Plant pathogenic bacteria | Reference(s) |
| Aminocoumarin | Novobiocin | Italy | *Xylella fastidiosa* | (Bleve et al., 2018) |
| Aminoglycosides | Amikacin | Italy | *X. fastidiosa* | (Bleve et al., 2018) |
|  | Gentamicin | Italy | *X. fastidiosa* | (Bleve et al., 2018) |
|  | Kanamycin | Italy | *X. fastidiosa* | (Bleve et al., 2018) |
|  | Kasugamycin | China, Italy, Pakistan | *Ralstonia solanacearum*, *Xanthomonas oryzae* pv*. oryzae*, *X. fastidiosa* | (Zhao et al., 2015; Bleve et al., 2018; Nasir et al., 2019) |
|  | Neomycin | Italy | *X. fastidiosa* | (Bleve et al., 2018) |
|  | Streptomycin | China, India, Italy, Pakistan | *R. solanacearum*, *Pectobacterium carotovorum*, *X. oryzae* pv. *oryzae*, *X. fastidiosa* | (Wang et al., 2015; Zhao et al., 2015; Kenganal et al., 2017; Bleve et al., 2018; Nasir et al., 2019) |
|  | Tobramycin | Italy | *X. fastidiosa* | (Bleve et al., 2018) |
| Cycloserine | Cycloserine (D) | Italy | *X. fastidiosa* | (Bleve et al., 2018) |
| Glycopeptides | Teicoplanin | Italy | *X. fastidiosa* | (Bleve et al., 2018) |
|  | Vancomycin | Italy | *X. fastidiosa* | (Bleve et al., 2018) |
| Lincosamides | Lincomycin | Italy | *X. fastidiosa* | (Bleve et al., 2018) |
| Macrolides | Spiramycin | Italy | *X. fastidiosa* | (Bleve et al., 2018) |
|  | Tetramycin | China | *Pseudomonas syringae* pv. *actinidiae* | (Wang et al., 2021) |
| Phenicols | Chloramphenicol | Italy | *X. fastidiosa* | (Bleve et al., 2018) |
| Polyene antifungal drug | Nystatin | Italy | *X. fastidiosa* | (Bleve et al., 2018) |
| Polymyxins | Polymyxin B | Italy | *X. fastidiosa* | (Bleve et al., 2018) |
| Polypeptides | Tyrothricin | Italy | *X. fastidiosa* | (Bleve et al., 2018) |
| Quinolones | Nalidixic acid | Italy | *X. fastidiosa* | (Bleve et al., 2018) |
| Rifamycin | Rifampicin | Italy, Pakistan | *Candidatus* Liberibacter sp., *X. fastidiosa* | (Bleve et al., 2018; Hussain et al., 2019) |
| Sulfonamides | Sulphapyridine | Italy | *X. fastidiosa* | (Bleve et al., 2018) |
| Tetracyclines | Doxycycline | Italy | *X. fastidiosa* | (Bleve et al., 2018) |
|  | Oxytetracycline | India, Italy | *X. oryzae* pv. *oryzae*, *X. fastidiosa* | (Bleve et al., 2018; Deep et al., 2020) |
|  | Tetracycline | India, Italy | *X. oryzae* pv. *oryzae*, *X. fastidiosa* | (Bleve et al., 2018; Deep et al., 2020) |
| β-lactams | Ampicillin | Italy, Pakistan | *Ca.* Liberibacter sp., *X. fastidiosa* | (Bleve et al., 2018; Hussain et al., 2019) |
|  | Carbenicillin | Italy | *X. fastidiosa* | (Bleve et al., 2018) |
|  | Cefalexin | Italy, Pakistan | *Ca.* Liberibacter sp., *X. fastidiosa* | (Bleve et al., 2018; Hussain et al., 2019) |
|  | Ceftriaxone | Italy | *X. fastidiosa* | (Bleve et al., 2018) |
|  | Cephaloglycin | Italy | *X. fastidiosa* | (Bleve et al., 2018) |
|  | Cephaloridine | Italy | *X. fastidiosa* | (Bleve et al., 2018) |
|  | Cephalotin | Italy | *X. fastidiosa* | (Bleve et al., 2018) |
|  | Penicillins (notably G, V) | Italy, USA | *Ca.* Liberibacter sp., *X. fastidiosa* | (Shin et al., 2016; Bleve et al., 2018; Killiny et al., 2019; McVay et al., 2019) |
| Combination | Plantomycin  (Streptomycin + tetracycline) | India | *X. oryzae* pv. *oryzae* | (Deep et al., 2020) |
| Combination | Streptocycline  (Streptomycin + tetracycline) | India | *R. solanacearum, Xanthomonas axonopodis* pv. c*ymopsidis, X. axonopodis* pv. *punicae*, *X. oryzae* pv. o*ryzae* | (Ambadkar et al., 2015; Deep et al., 2020; Singh, 2020; Godara and Singh, 2021) |

Ambadkar, C. V., Dhawan, A. S., and Shinde, V. N. (2015). Integrated management of bacterial blight disease (oily spot) of pomegranate caused by *Xanthomonas axonopodis* pv. *punicae*. *Int. J. Plant Sci.* 10, 19–23. doi: 10.15740/has/ijps/10.1/19-23.

Bleve, G., Gallo, A., Altomare, C., Vurro, M., Maiorano, G., Cardinali, A., et al. (2018). *In vitro* activity of antimicrobial compounds against *Xylella fastidiosa*, the causal agent of the olive quick decline syndrome in Apulia (Italy). *FEMS Microbiol. Lett.* 365, 1–10. doi: 10.1093/femsle/fnx281.

Deep, S., Prasad, D., and Sarkhel, S. (2020). Evaluation of antibiotics, fungitoxicants and botanicals against *Xanthomonas oryzae* pv. *oryzae*, A cause of bacterial leaf blight of rice. *Int. J. Plant Prot.* 13, 1–8. doi: 10.15740/has/ijpp/13.1/1-8.

Godara, S. L., and Singh, N. (2021). Management of bacterial leaf blight (*Xanthomonas axonopodis* pv. *cymopsidis*) of clusterbean in Rajasthan. *Indian Phytopathol.* 74, 223–227. doi: 10.1007/s42360-020-00299-8.

Hussain, S., Rao, M. J., Anjum, M. A., Ejaz, S., Umar, U. ud D., Ali, M. A., et al. (2019). Effect of different combinations of antibiotics on fruit quality and antioxidant defense system in Huanglongbing infected Kinnow orchards. *AMB Express* 9. doi: 10.1186/s13568-019-0871-9.

Kenganal, M., Nimbaragi, Y. A., and Guruprasd, G. S. (2017). Management of soft rot of banana caused by *Erwinia carotovora* subsp. *carotovora*. *Int. J. Plant Prot.* 10, 381–385. doi: 10.15740/has/ijpp/10.2/381-385.

Killiny, N., Gonzalez-Blanco, P., Santos-Ortega, Y., Al-Rimawi, F., Levy, A., Hijaz, F., et al. (2019). Tracing penicillin movement in citrus plants using fluorescence-labeled penicillin. *Antibiotics* 8, 1–9. doi: 10.3390/antibiotics8040262.

McVay, J., Sun, X., Jones, D., Urbina, H., Aldeek, F., Cook, J. M., et al. (2019). Limited persistence of residues and metabolites in fruit and juice following penicillin trunk infusion in citrus affected by Huanglongbing. *Crop Prot.* 125, 104753. doi: 10.1016/j.cropro.2019.03.001.

Nasir, M., Iqbal, B., Hussain, M., Mustafa, A., and Ayub, M. (2019). Chemical management of bacterial leaf blight. *J Agric. Res.* 57, 93–98.

Shin, K., Ascunce, M. S., Narouei-Khandan, H. A., Sun, X., Jones, D., Kolawole, O. O., et al. (2016). Effects and side effects of penicillin injection in huanglongbing affected grapefruit trees. *Crop Prot.* 90, 106–116. doi: 10.1016/j.cropro.2016.08.025.

Singh, R. (2020). Integrated management of bacterial wilt of ginger incited by *Ralstonia solanacearum*. *Int. J. Plant Sci.* 15, 86–91. doi: 10.15740/has/ijps/15.2/86-91.

Wang, H., Wang, J., Xia, H., Huang, Y., Wang, M., Jia, M., et al. (2015). Sensitivities of *Ralstonia solanacearum* to streptomycin, calcium oxide, mancozeb and synthetic fertilizer. *Plant Pathol. J.* 14, 13–22. doi: 10.3923/ppj.2015.13.22.

Wang, Q., Zhang, C., Long, Y., Wu, X., Su, Y., Lei, Y., et al. (2021). Bioactivity and control efficacy of the novel antibiotic tetramycin against various kiwifruit diseases. *Antibiotics* 10, 1–11. doi: 10.3390/antibiotics10030289.

Zhao, Z., Yan, W., Chen, Y., Xiao, T., and Xiao, M. (2015). Toxicity of several bactericides against bacterial wilt of ginger. *Guizhou Agric. Sci.* 43, 76–78.
